# Ultrasonographic Assessment of Indian Patients With Plantar Fasciitis and Its Clinical Correlation: A Prospective Observational Study

**DOI:** 10.7759/cureus.35764

**Published:** 2023-03-04

**Authors:** Suhas Aradhya BM, Vivek Tiwari, Ashwini M Bakde, Samir Dwidmuthe, Mainak Roy

**Affiliations:** 1 Orthopedics, All India Institute of Medical Sciences, Nagpur, IND; 2 Radiodiagnosis, All India Institute of Medical Sciences, Nagpur, IND

**Keywords:** ultrasonography, heel fat pad, body mass index, plantar fasciitis, ultrasound, indian patients, footwear

## Abstract

Introduction: Plantar fasciitis is a debilitating clinical condition and is one of the most common causes of heel pain. The risk factors include frequent and prolonged running, obesity, a sedentary lifestyle, work-related weight bearing, and inappropriate footwear. Ultrasonography being a non-invasive, cost-effective, and easily available modality is a useful adjunct in the diagnosis.

Methods: A prospective observational study was conducted among 30 patients with unilateral plantar fasciitis. The diagnosis was based on history and examination. Heel pad thickness and plantar fascia thickness were recorded using ultrasonography.

Results: The ultrasonography results showed increased plantar fascia and heel pad thickness in the affected limb with plantar fasciitis than the normal one (p<0.001). The BMI was positively correlated with the heel pad thickness (p<0.05). The receiver operating characteristic (ROC) curve showed 90% sensitivity and 60% specificity for heel pad thickness (p<0.001).

Conclusions: Ultrasonography is a sensitive and specific tool to identify patients with plantar fasciitis.

## Introduction

Heel pain is a common problem encountered in orthopedic practice and treatment is mostly disappointing. The pain begins gradually and may become severe, continuous, or intermittent [1]. Plantar fasciitis is the most common cause of heel pain, affecting adults with both active and sedentary lifestyles [2].

The plantar fascia is a multilayered thickened fibrous sheet of connective tissue originating on the plantar surface of the posteromedial calcaneal tuberosity. It provides support to the longitudinal arch of the foot and acts as a dynamic shock absorber during foot strikes [3]. Plantar fasciitis is the non-inflammatory, degenerative process of the plantar fascia. In the presence of aggravating factors, there is tension along the fascia due to repeated strain, leading to micro tears and subsequent inflammation at its insertion. It is more likely to occur in women aged 40 to 60 years [4].

The persistence of these risk factors inhibits the normal repair process leading to collagen degeneration and structural changes in the plantar fascia. The thickened plantar fascia causes a decrease in elasticity and decreases in the shock-absorbing capabilities of the fascia. The main risk factors for plantar fasciitis are: occupations that require prolonged standing and walking, excessive running, sedentary lifestyle, excessive foot pronation (pes planus), high arch (pes cavus), leg length discrepancy, obesity, and tightness of the Achilles tendon and intrinsic foot muscles [5]. There is conflicting evidence of an association between heel pad thickness and plantar heel pain [6].

When diagnosed and treated properly, 80% of patients with plantar fasciitis improve within a year. It is predominantly a clinical diagnosis. Main clinical symptoms are stabbing, non-radiating pain in the proximal medial-plantar surface of the foot. On examination, there is tenderness at the anteromedial calcaneus. The windlass test is useful in diagnosis. A positive result is heel pain reproduced by forced dorsiflexion of the toes at the metatarsophalangeal joints with the ankle stabilized [7].

Currently, there is no objective, reliable diagnostic test for plantar fasciitis [4]. Although not routinely needed initially, imaging can aid in confirming recalcitrant plantar fasciitis or rule out other heel pathology [5,8]. Plain radiography helps identify the bony lesions of the foot. Sub calcaneal spur found on lateral foot radiography does not support the diagnosis of plantar fasciitis and can also be found in patients without plantar fasciitis [4,8]. Although people with chronic heel pain are more likely to have a bone spur, the spur will remain after symptoms resolve [7].

Ultrasonography (USG) is a reasonable and accurate diagnostic tool in ruling out soft tissue pathology of the heel. An USG is a reliable alternative to MRI for evaluating heel pain [4,7,8]. Studies showed similar accuracy and effectiveness of USG in morphological assessment and diagnosis of plantar fasciitis compared to MRI [9]. The USG is usually preferred because of its certain advantages over MRI such as non-invasiveness, relatively inexpensive modality with excellent spatial resolution, and being well tolerated by patients. Real-time high-resolution USG has been proven to be a valuable technique [3]. Thickened plantar fascia and tissue abnormalities, hypoechoic changes, peri fascial fluid collections, and bony spur have been reported in USG [3-5].

Despite the high prevalence of around 4% to 7% and the impact on those who experience it, the etiology of plantar heel pain is not well understood, which leads to uncertainty regarding the most effective intervention strategy [2,10]. Although there have been studies reporting the various USG findings in plantar fasciitis from the western world, there is a dearth of such studies in Indian patients [11,12]. Hence this study was conducted to assess the demographic profile of Indian patients with plantar fasciitis, to evaluate the USG findings of heels in Indian patients with plantar fasciitis, and to correlate the various clinical and demographic factors with the USG findings.

## Materials and methods

This is a prospective observational study conducted at a tertiary-care hospital in central India. Thirty patients with unilateral plantar fasciitis were enrolled. Those with a history of any recent trauma to the foot, any foot surgery, history of receiving steroid/platelet-rich plasma injection in the heel, history of systemic inflammatory disease including rheumatoid arthritis, diabetes mellitus, any spine pathology with radiation of pain to lower limb or connective tissue disorders were excluded from the study. A simple random sampling technique was used to select the study participants.

**Figure 1:**
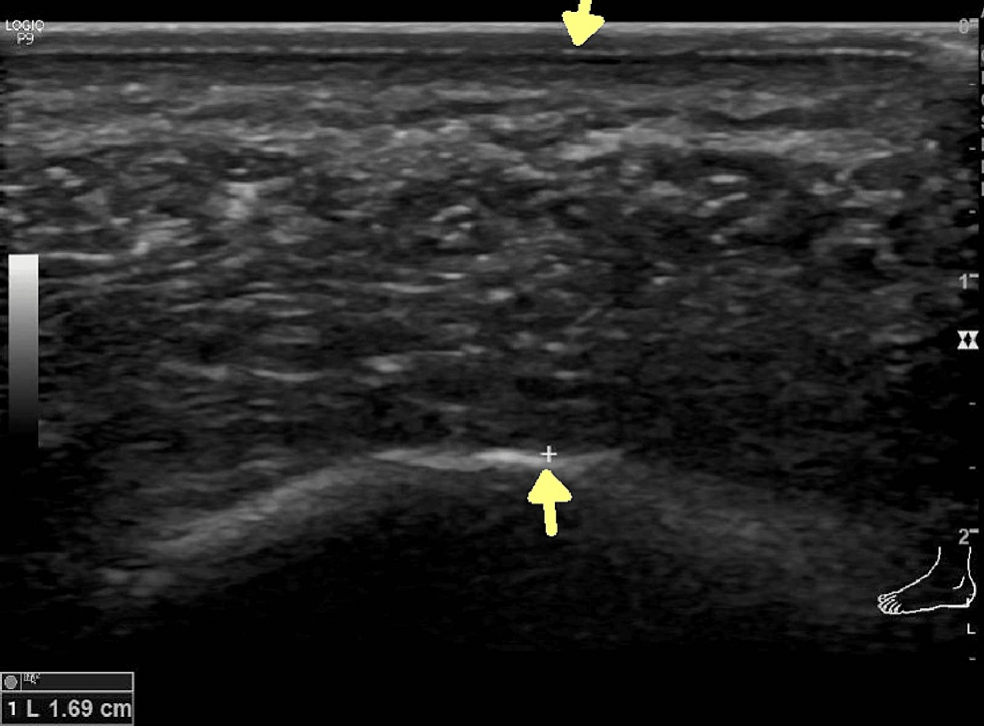
Ultrasonographic picture showing normal heel pad thickness (yellow arrows)

Data collection was started after obtaining clearance from the Institutional Ethics Committee of All India Medical Sciences (AIIMS), Nagpur, India (approval no. IEC/Pharmac/58/20). Informed consent was taken from study participants. After explaining the purpose of the study, demographic and clinical information was collected including age, sex, BMI, occupation, duration of symptoms, and footwear preferences. Patients were classified into active or sedentary lifestyles based on their occupation. Those having to sit for prolonged hours of work on chairs requiring little to no physical movement were grouped into a sedentary lifestyle, while those having occupations where regular physical activity was involved were grouped into an active lifestyle. The diagnosis of plantar fasciitis was made clinically based on history and examination findings. The clinical severity of pain was assessed using the visual analog score (VAS) on a scale of 0-10. Weight-bearing anteroposterior and lateral X-rays of both feet were obtained. The angle of the medial arch (AMA) and first metatarsophalangeal angle (FMTPA) were measured. Any associated findings like calcaneal spur were also recorded. Ultrasonography of both heels was done on a GE ultrasound machine LOGIQ-P9 (GE Healthcare, Chicago, IL, USA) with a 12 MHZ linear probe. The patient was asked to lie down in a prone position with the foot resting on the toe. Heel pad thickness and plantar fascia thickness were measured on both sides of the heel without applying any pressure on the probe. Heel pad thickness was measured from the sole to the surface of the calcaneum, and normally it should be less than 2.2 mm (Figure 1).

**Figure 2:**
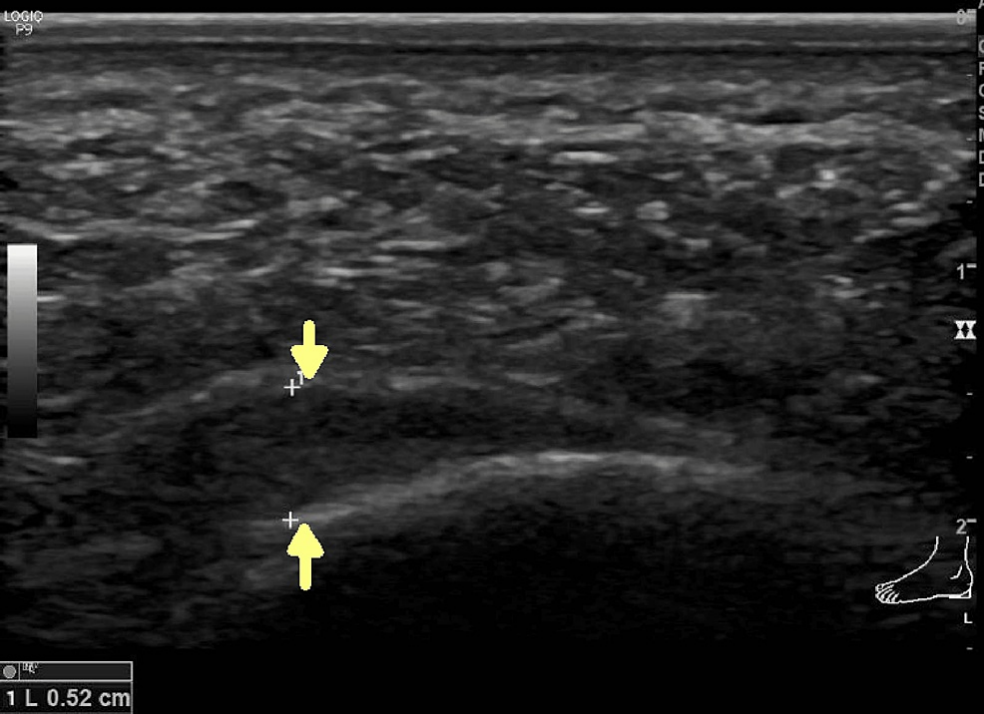
Ultrasonographic picture showing the thickened and hypoechoic plantar fascia

Plantar fascia thickness was measured within 2 cm from its attachment to the calcaneum, and normally it should be less than 4 mm (Figure 2).

Data analysis was done using SPSS version 20 (IBM Corp., Armonk, NY, USA). Descriptive analysis (means, standard deviations, and percentages/proportions) was done for demographic details. Student’s t-test was used to compare AMA, FMTPA, plantar fascia thickness, and heel pad thickness between symptomatic and unaffected feet. Pearson correlation analysis was done to correlate various clinico-demographic factors with the X-ray and USG measurements. A p-value of <0.05 was considered significant.

## Results

Among the 30 participants, 12 (40%) were males and 18 (60%) were females. The minimum age of the participants was 27 years and the maximum was 55 years. The mean age was 39.8 ± 8.8 years. The age distribution of the study participants is tabulated in Table 1.

**Table 1:** Age distribution of the participants (n=30)

Age	Frequency (N)	Percentage (%)
<40 years	17	56.7
>40 years	13	43.3
Total	30	100

The majority of the study participants had a sedentary lifestyle (22 (73.3%)) whereas eight (26.7%) had an active lifestyle. The occupational activity of the participants is tabulated in Table 2.

**Table 2:** Occupational activity of the participants (n=30)

Occupation	Frequency (N)	Percentage (%)
Fieldwork	8	26.7
Office work	8	26.7
Housewife	12	40.0
Teacher	1	3.3
Security	1	3.3
Total	30	100

**Figure 3:**
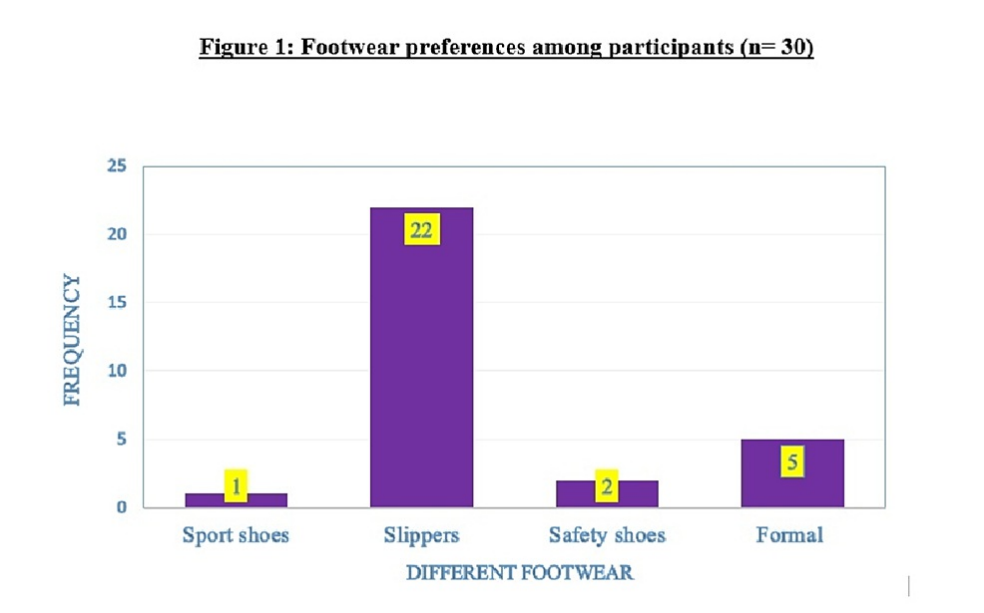
Footwear preferences among participants (n=30)

According to the Indian/Asian classification of BMI, six (20%) were of normal BMI, five (16.7%) were overweight, and 19 (63.3%) were obese. The mean BMI was 27 ± 4.17 kg/m^2^. The majority of the participants were using slippers (73.3%) followed by formal shoes (16.7%), safety shoes (6.7%), and sports shoes (3.3%) (Figure 3).

The mean duration of symptoms was 105 ± 56.6 days. Around 14 (46.7%) had right-sided plantar fasciitis, and 16 (53.3%) had left-sided plantar fasciitis. The mean VAS score was found to be 6.4 ± 1.4. With the categorization of the VAS score, it was found that none had mild pain. The majority had moderate pain (60%) followed by severe pain (40%). Calcaneal spurs were present among the majority of patients: 25 (83.3%) in the affected leg, and 16 (53.3%) in the unaffected leg.

Though in the X-rays, the AMA and FMTPA were higher in the leg with plantar fasciitis than in the unaffected leg, it was not statistically significant. The plantar fascia thickness was more in the leg with plantar fasciitis when compared with the normal unaffected leg. Heel pad thickness was also increased in the leg with plantar fasciitis than in the normal leg. Both were found statistically significant (p<0.001) (Table 3).

**Table 3:** Comparison of radiological features in the affected and unaffected leg (n=30) PF: Plantar fasciitis, SD: Standard deviation, AMA: Angle of the medial arch, FMTP: First metatarsophalangeal angle

S. No.	Radiological measurement	Leg with PF (Mean & SD)	Unaffected feet (Mean & SD)	p-value
1	AMA	132^0^ ± 2.1	130.2^0^ ± 11.8	0.075
2	FMTPA	12.2^0 ^± 6	10.8^0^ ± 4.2	0.156
3	PF thickness (mm)	5.07 ± 0.9	3.11 ± 0.4	<0.001
4	Heel pad thickness (mm)	19.4 ± 1.9	16.5 ± 2.4	<0.001

Table 4 shows the correlation of USG plantar fascia thickness with the demographic and clinical features. Pearson’s correlation value indicates that age and BMI were negatively correlated with plantar fascia thickness in the affected leg, but they were statistically not significant. The duration of the symptoms and the VAS score were positively correlated with the plantar fascia thickness in the affected leg but they too were statistically not significant.

**Table 4:** Correlation of ultrasonographic plantar fascia thickness with the demographic and clinical features (n=30) PF: Plantar fasciitis, VAS: Visual analog scale

Demographic/clinical features	Ultrasonographic PF thickness			
	Affected leg		Unaffected leg	
	Pearson’s correlation (r) value	p-value	Pearson’s correlation (r) value	p-value
Age	-0.024	0.902	+0.283	0.130
BMI	-0.011	0.954	-0.230	0.222
Duration of the symptoms	+0.158	0.404	+0.404	0.027
VAS score	+0.053	0.780	+0.071	0.711

Table 5 shows the correlation of USG heel pad thickness with the demographic and clinical features. In the affected leg, age, duration of symptoms, and VAS score were negatively correlated but were statistically not significant. The BMI was positively correlated with the heel pad thickness and was found statistically significant.

**Table 5:** Correlation of ultrasonographic heel pad thickness with the demographic and clinical features (n=30) VAS: Visual analog scale

Demographic/clinical features	Ultrasonographic heel pad thickness			
	Affected leg		Unaffected leg	
	Pearson’s correlation (r) value	p-value	Pearson’s correlation (r) value	p-value
Age	-0.199	0.291	+0.239	0.203
BMI	+0.404	0.027	+0.184	0.330
Duration of the symptoms	-0.273	0.144	+0.693	0.000
VAS score	-0.091	0.631	+0.122	0.521

**Figure 4:**
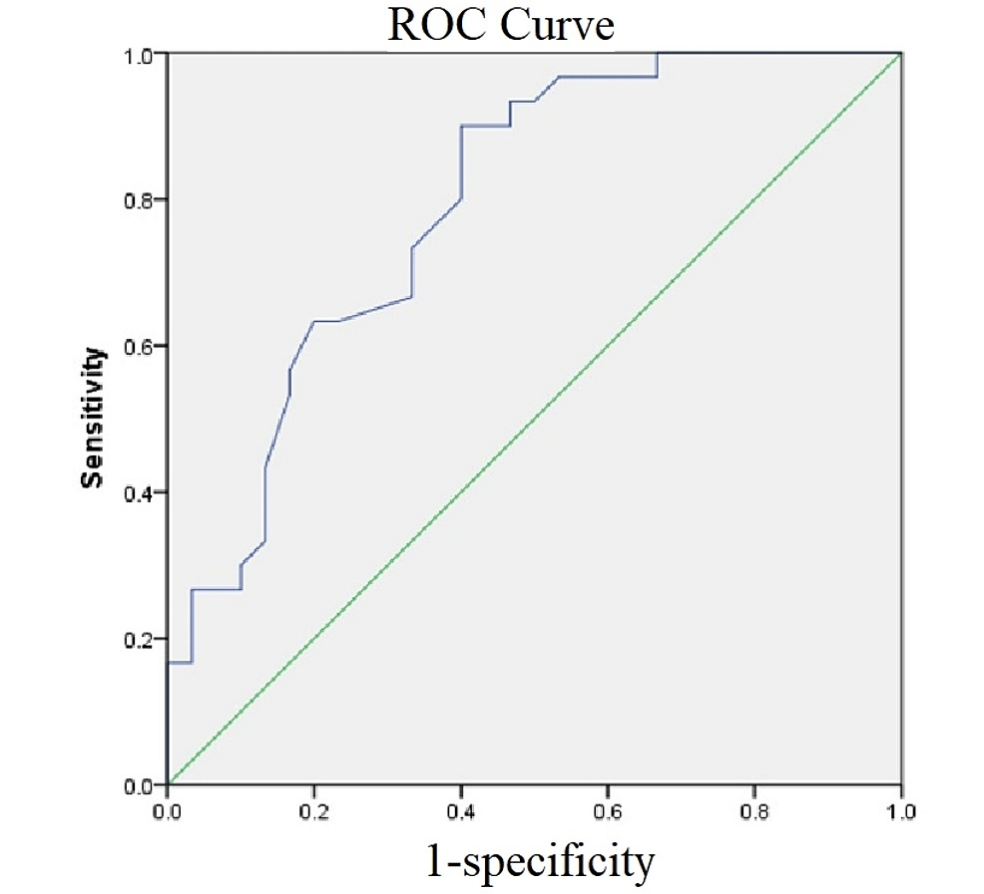
The ROC curve for heel pad thickness in ultrasonography ROC: Receiver operating characteristic

The receiver operating characteristic (ROC) curve was used to test the sensitivity and specificity of the USG features with clinically diagnosed plantar fasciitis patients. The ROC curve showed that USG has good sensitivity and specificity for diagnosing plantar fasciitis with respect to heel pad thickness, with the area under the curve being 0.8 and a sensitivity of 90% and 60% specificity when the cut-off was 17.45. It was statistically highly significant (p<0.001) (Figure 4).

## Discussion

Almost 15% of the patients with heel complaints have plantar fasciitis [13]. However, data on the demographic, clinical as well as diagnostic aspect is very sparse in the Indian population. It is important to have an early diagnosis to rule out other causes of heel pain such as tendonitis, arthritis, nerve irritation, or bone pathologies. Diagnosis of plantar fasciitis is largely done by the patient’s symptoms and signs. A reliable and cost-effective diagnostic test is extremely helpful to diagnose plantar fasciitis.

In this study, 30 patients who were diagnosed clinically with unilateral plantar fasciitis were enrolled and their demographic and clinical parameters were correlated with the USG findings. According to Barrett et al., plantar fasciitis is more common in women [13]. In this study too, we found females were affected more than males. The mean age of occurrence of plantar fasciitis was found around 40 years which is similar to the study by Khatiwada et al. where the mean age was 40 to 50 years and females were affected more [3]. The majority (73.3%) of the study participants had a sedentary lifestyle in our study. Similarly, in the study by Belhan et al., 54% of the patients had a sedentary lifestyle [5]. It is known that the risk of developing plantar fasciitis is higher in individuals with a sedentary lifestyle [5]. In this study, the majority of patients were obese (63.3%). The mean BMI was 27 ± 4.2 kg/m^2 ^similar to the BMI in a study by Belhan et al. where it was 28.7 kg/m^2^ [5]. It has been reported by Rajput et al. that footwear is involved in the etiology of plantar fasciitis [14]. Improper footwear preferences can trigger the occurrence of plantar fasciitis, aggravate the existing condition, or reduce the response to treatment [15,16]. Shoes that reduce pronation and partially increase heel height are the preferred footwear modifications in plantar fasciitis [17]. In the present study, the majority of the participants were using slippers (73.3%).

Calcaneal spurs were present among the majority of patients: 25 (83.3%) in the affected leg and 16 (53.3%) in the unaffected leg. Similarly, the calcaneal spur was observed in 66% of the heels with pain and in 22% of the heels without pain in a previous study [5].

The radiological features were compared between the affected and unaffected legs to find out the efficacy of the tests to identify plantar fasciitis. Though in X-rays the AMA and FMTPA were higher in the leg with plantar fasciitis than in the unaffected leg, it was not statistically significant. On the other hand, in a previous study, it was observed that the mean AMA in the heel with pain was significantly higher than that of the heel without pain (p<0.05) [5].

The plantar fascia thickness was more in the leg with plantar fasciitis when compared with the normal unaffected leg. Heel pad thickness was also increased in the leg with plantar fasciitis than in the normal leg. Both were found statistically significant (p<0.001). The mean plantar fascia thickness in the affected leg was 5.07 ± 0.9 mm and the finding is consistent with that of Khatiwada et al. (4.63 ± 0.55mm) [3]. The study by Karabay et al. also found similar results, where the mean plantar fascia thickness in the affected leg was 4.79 mm but no significant difference was found between heel pads of the diseased and healthy feet [4]. Ozdemir et al. also found a greater amount of sub calcaneal fat pad in patients afflicted with plantar pain [1]. According to the study by Wall et al., a plantar fascia thickness of more than 4 mm if associated with inflammatory changes and would be consistent with plantar fasciitis [17]. Tsai et al. also chose a thickness of 4 mm as the cut-off point to distinguish normal fascia from inflammatory fascia [18].

Pearson’s correlation test was done to find out the correlation between various clinical parameters and USG plantar fascia thickness. It was found that as age and BMI increased, the plantar fascia thickness reduced (negative correlation), contradicting the findings by Khatiwada et al. [3]. Also, as the duration of symptoms and pain severity increased, the plantar fascia thickness increased; however, it was not statistically significant.

We found that as BMI increases, the heel pad thickness will also increase. Similarly, the results of the study by Rome et al. indicate that as BMI increased, there was a concomitant increase in heel pad thickness [6]. Elderly people have a thicker and stiffer heel fat pad compared to young people. This greatly reduces the shock absorption capacity of the heel fat pad and, as a result, makes it more susceptible to injury. The intrinsic and extrinsic muscles of the foot weaken with aging and the medial longitudinal arch cannot provide adequate support, consequently, the foot arch height decreases [6].

The USG method is one of the inexpensive tests to diagnose plantar fasciitis. To check its efficacy concerning identifying the true positive cases among all cases (sensitivity) and to detect all true negatives among those who do not have the disease (specificity), the ROC curve was created. The curve demonstrated that USG has good sensitivity and specificity for diagnosing plantar fasciitis with respect to heel pad thickness, with the area under the curve being 0.8 (excellent). A sensitivity of 90% and 60% specificity was seen when the cut-off was 17.45. It was statistically highly significant (p<0.001). In a study by Tsai et al., the sensitivity of ultrasound in detecting plantar fasciitis was 91.9% and the specificity was 90.5% [18].

There were some limitations in our study including the relatively small sample size, single institutional study, and observational nature of the study. However, it provides useful information regarding the radiological findings in Indian patients with plantar fasciitis and their clinical correlation, which will help plan future large-scale studies on plantar fasciitis in India.

## Conclusions

This study concludes that the USG features in patients with plantar fasciitis were statistically significant concerning the plantar fascia thickness and heel pad thickness. The BMI was positively correlated with heel pad thickness. It also confirms that USG is a sensitive and specific measure to find out the increased heel pad thickness in patients with plantar fasciitis.
